# Final 2013 Reports of Nationally Notifiable Infectious Diseases

**Published:** 2014-08-15

Table 2 listed on pages 703–15 summarizes finalized data, as of June 30, 2014, from the National Notifiable Diseases Surveillance System (NNDSS) for 2013. These data will be published in more detail next year in the *Summary of Notifiable Diseases — United States, 2013* (1). Because no cases were reported in the United States during 2013, the following diseases do not appear in these early release tables: anthrax; diphtheria; eastern equine encephalitis, nonneuroinvasive disease; poliovirus infection, nonparalytic; severe acute respiratory syndrome–associated coronavirus disease (SARS-CoV); smallpox; St. Louis encephalitis, nonneuroinvasive disease; western equine encephalitis, neuroinvasive and nonneuroinvasive disease; yellow fever; and viral hemorrhagic fevers. Data on chronic hepatitis B and hepatitis C virus infection (past or present) are not included because they are undergoing data quality review. Data for human immunodeficiency virus (HIV) diagnoses do not appear because CDC is transitioning to a new system for processing national HIV surveillance data, and will be published in the *Summary of Notifiable Diseases — United States, 2013*.

Policies for reporting NNDSS data to CDC can vary by disease or reporting jurisdiction depending on case status classification (i.e., confirmed, probable, or suspected). The publication criteria used for the 2013 finalized tables are listed in the “Print Criteria” column of the NNDSS event code list, available at http://wwwn.cdc.gov/nndss/document/nndss_event_code_list_2013_revised.pdf. In addition, only cases from reporting jurisdictions where the nationally notifiable disease is reportable are published. The NNDSS website is updated annually to include the latest national surveillance case definitions approved by the Council of State and Territorial Epidemiologists for classifying and enumerating cases of nationally notifiable infectious diseases.

Population estimates are from the National Center for Health Statistics postcensal estimates of the resident population of the United States for July 1, 2010–July 1, 2012, by year, county, single year of age (0 to ≥85 years), bridged-race (white, black or African American, American Indian or Alaska Native, Asian or Pacific Islander), Hispanic ethnicity (not Hispanic or Latino, Hispanic or Latino), and sex (vintage 2012), prepared under a collaborative arrangement with the U.S. Census Bureau. Population estimates for states are available at http://www.cdc.gov/nchs/nvss/bridged_race/data_documentation.htm#vintage2012. Population estimates for territories are 2012 estimates from the U.S. Census Bureau (2).

## Figures and Tables

**TABLE 2:** Reported cases of notifiable diseases,* by geographic division and area — United States, 2013

Area	Total resident population (in thousands)	Arboviruses†							

		California serogroup§		Eastern equine encephalitis	Powassan		St. Louis encephalitis	West Nile	
				
		Neuro-invasive	Nonneuro-invasive	Neuro-invasive	Neuro-invasive	Nonneuro-invasive	Neuro-invasive	Neuro-invasive	Nonneuro-invasive
**United States**	313,875	95	17	8	12	3	1	1,267	1,202
**New England**	14,564	3	—	2	3	—	—	11	5
Connecticut	3,592	—	—	1	—	—	—	1	3
Maine	1,329	—	—	—	1	—	—	—	—
Massachusetts	6,645	1	—	1	1	—	—	7	1
New Hampshire	1,322	1	—	—	1	—	—	1	—
Rhode Island	1,050	1	—	—	—	—	—	1	—
Vermont	626	—	—	—	—	—	—	1	1
**Mid. Atlantic**	41,208	3	1	—	5	1	—	34	21
New Jersey	8,868	—	—	—	1	—	—	10	2
New York (Upstate)	11,232	3	—	—	4	1	—	10	12
New York City	8,344	—	—	—	—	—	—	8	2
Pennsylvania	12,764	—	1	—	—	—	—	6	5
**E.N. Central**	46,567	29	10	—	3	2	—	167	54
Illinois	12,868	—	—	—	—	—	—	86	31
Indiana	6,538	1	—	—	—	—	—	19	4
Michigan	9,883	—	—	—	—	—	—	24	12
Ohio	11,553	14	2	—	—	—	—	21	3
Wisconsin	5,725	14	8	—	3	2	—	17	4
**W.N. Central**	20,755	5	1	—	1	—	—	288	455
Iowa	3,075	—	—	—	—	—	—	24	20
Kansas	2,885	—	—	—	—	—	—	34	57
Minnesota	5,380	5	1	—	1	—	—	31	48
Missouri	6,025	—	—	—	—	—	—	24	5
Nebraska	1,855	—	—	—	—	—	—	54	172
North Dakota	701	—	—	—	—	—	—	64	61
South Dakota	834	—	—	—	—	—	—	57	92
**S. Atlantic**	61,187	27	2	5	—	—	—	36	18
Delaware	917	—	—	—	—	—	—	3	—
District of Columbia	633	—	—	—	—	—	—	—	1
Florida	19,321	—	—	3	—	—	—	5	2
Georgia	9,916	1	1	1	—	—	—	4	6
Maryland	5,885	—	—	—	—	—	—	11	5
North Carolina	9,748	13	—	1	—	—	—	3	—
South Carolina	4,723	1	—	—	—	—	—	3	4
Virginia	8,187	2	—	—	—	—	—	6	—
West Virginia	1,857	10	1	—	—	—	—	1	—
**E.S. Central**	18,639	27	1	—	—	—	—	48	33
Alabama	4,818	2	—	—	—	—	—	3	6
Kentucky	4,380	—	—	—	—	—	—	1	2
Mississippi	2,986	2	1	—	—	—	—	27	18
Tennessee	6,455	23	—	—	—	—	—	17	7
**W.S. Central**	37,429	—	—	1	—	—	1	223	121
Arkansas	2,950	—	—	1	—	—	—	16	2
Louisiana	4,602	—	—	—	—	—	—	34	20
Oklahoma	3,816	—	—	—	—	—	—	60	29
Texas	26,061	—	—	—	—	—	1	113	70
**Mountain**	22,611	—	2	—	—	—	—	216	343
Arizona	6,551	—	—	—	—	—	—	50	12
Colorado	5,189	—	—	—	—	—	—	90	232
Idaho	1,596	—	1	—	—	—	—	14	26
Montana	1,005	—	—	—	—	—	—	10	28
Nevada	2,754	—	1	—	—	—	—	8	3
New Mexico	2,084	—	—	—	—	—	—	24	14
Utah	2,855	—	—	—	—	—	—	4	3
Wyoming	577	—	—	—	—	—	—	16	25
**Pacific**	50,915	1	—	—	—	—	—	244	152
Alaska	730	—	—	—	—	—	—	—	—
California	38,000	—	—	—	—	—	—	237	142
Hawaii	1,390	—	—	—	—	—	—	—	—
Oregon	3,900	1	—	—	—	—	—	7	9
Washington	6,895	—	—	—	—	—	—	—	1

**Territories**									
American Samoa	55	—	—	—	—	—	—	—	—
C.N.M.I.	51	—	—	—	—	—	—	—	—
Guam	160	—	—	—	—	—	—	—	—
Puerto Rico	3,673	—	—	—	—	—	—	—	—
U.S. Virgin Islands	105	—	—	—	—	—	—	—	—

	Babesiosis			Botulism					
				
Area	Total	Confirmed	Probable	Total	Foodborne	Infant	Other†	Brucellosis	Chancroid§
**United States**	1,792	1,480	312	152	4	136	12	99	10
**New England**	920	812	108	1	1	—	—	1	2
Connecticut	289	249	40	—	—	—	—	1	—
Maine	36	30	6	—	—	—	—	—	—
Massachusetts	425	396	29	1	1	—	—	—	2
New Hampshire	22	18	4	—	—	—	—	—	—
Rhode Island	142	117	25	—	—	—	—	—	—
Vermont	6	2	4	—	—	—	—	—	—
**Mid. Atlantic**	705	553	152	28	—	27	1	8	—
New Jersey	171	137	34	4	—	4	—	—	—
New York (Upstate)	459	348	111	1	—	1	—	4	—
New York City	75	68	7	3	—	3	—	—	—
Pennsylvania	N	N	N	20	—	19	1	4	—
**E.N. Central**	81	68	13	6	—	6	—	13	—
Illinois	N	N	N	1	—	1	—	5	—
Indiana	1	—	1	—	—	—	—	1	—
Michigan	2	2	—	—	—	—	—	—	—
Ohio	N	N	N	5	—	5	—	2	—
Wisconsin	78	66	12	—	—	—	—	5	—
**W.N. Central**	67	34	33	6	—	6	—	9	—
Iowa	N	N	N	3	—	3	N	2	—
Kansas	N	N	N	1	—	1	—	—	—
Minnesota	64	32	32	—	—	—	—	1	—
Missouri	N	N	N	1	—	1	—	2	—
Nebraska	1	—	1	1	—	1	—	3	—
North Dakota	1	1	—	—	—	—	—	—	—
South Dakota	1	1	—	—	—	—	—	1	—
**S. Atlantic**	12	7	5	15	—	15	—	18	—
Delaware	2	2	—	3	—	3	—	—	—
District of Columbia	N	N	N	—	—	—	—	—	—
Florida	N	N	N	—	—	—	—	9	—
Georgia	N	N	N	—	—	—	—	5	—
Maryland	9	4	5	8	—	8	—	—	—
North Carolina	N	N	N	—	—	—	—	—	—
South Carolina	1	1	—	—	—	—	—	1	—
Virginia	N	N	N	3	—	3	—	3	—
West Virginia	—	—	—	1	—	1	—	—	—
**E.S. Central**	—	—	—	5	—	5	—	3	1
Alabama	—	—	—	—	—	—	—	1	1
Kentucky	N	N	N	2	—	2	—	1	—
Mississippi	N	N	N	2	—	2	—	—	—
Tennessee	—	—	—	1	—	1	—	1	—
**W.S. Central**	3	2	1	14	—	12	2	22	1
Arkansas	N	N	N	1	—	1	—	3	—
Louisiana	2	1	1	3	—	3	—	3	—
Oklahoma	N	N	N	1	—	1	—	5	—
Texas	1	1	—	9	—	7	2	11	1
**Mountain**	—	—	—	12	—	12	—	2	—
Arizona	N	N	N	1	—	1	—	1	—
Colorado	N	N	N	4	—	4	—	1	—
Idaho	N	N	N	1	—	1	—	—	—
Montana	—	—	—	—	—	—	—	—	—
Nevada	N	N	N	1	—	1	—	—	—
New Mexico	N	N	N	3	—	3	—	—	—
Utah	—	—	—	2	—	2	—	—	—
Wyoming	—	—	—	—	—	—	—		—
**Pacific**	4	4	—	65	3	53	9	23	6
Alaska	N	N	N	1	1	—	—	—	—
California	3	3	—	56	1	46	9	20	6
Hawaii	N	N	N	—	—	—	—	—	—
Oregon	—	—	—	4	1	3	—	2	—
Washington	1	1	—	4	—	4	—	1	—

**Territories**									
American Samoa	U	U	U	—	—	—	—	—	—
C.N.M.I.	—	—	—	—	—	—	—	—	—
Guam	—	—	—	—	—	—	—	—	—
Puerto Rico	N	N	N	—	—	—	—	—	—
U.S. Virgin Islands	N	N	N	—	—	—	—	—	—

Area	*Chlamydia trachomatis* infection†	Cholera	Coccidioidomycosis	Cryptosporidiosis			Cyclosporiasis

				Total	Confirmed	Probable	
**United States**	1,401,906	14	9,438	9,056	5,698	3,358	784
**New England**	48,696	4	1	277	256	21	9
Connecticut	12,775	1	N	36	36	—	3
Maine	3,438	—	N	35	25	10	N
Massachusetts	23,210	3	—	123	123	—	5
New Hampshire	3,119	—	1	45	34	11	1
Rhode Island	4,312	—	—	12	12	—	—
Vermont	1,842	—	N	26	26	—	N
**Mid. Atlantic**	176,186	1	—	844	636	208	34
New Jersey	28,327	1	N	70	69	1	13
New York (Upstate)	37,922	—	N	252	243	9	6
New York City	57,881	—	N	81	81	—	15
Pennsylvania	52,056	—	N	441	243	198	N
**E.N. Central**	213,348	1	28	1,524	1,085	439	56
Illinois	63,797	1	N	266	143	123	23
Indiana	28,023	—	N	139	107	32	1
Michigan	44,835	—	16	270	234	36	2
Ohio	53,121	—	7	372	124	248	7
Wisconsin	23,572	—	5	477	477	—	23
**W.N. Central**	82,195	2	91	2,547	1,083	1,464	252
Iowa	10,953	1	N	1,505	498	1,007	148
Kansas	11,012	—	N	99	60	39	4
Minnesota	18,742	1	64	324	224	100	3
Missouri	27,328	—	17	210	97	113	5
Nebraska	7,301	—	1	151	103	48	91
North Dakota	2,932	—	9	84	63	21	N
South Dakota	3,927	—	N	174	38	136	1
**S. Atlantic**	282,067	5	8	1,181	716	465	57
Delaware	5,213	1	1	16	8	8	—
District of Columbia	6,414	—	1	15	14	1	N
Florida	80,182	4	N	409	201	208	47
Georgia	51,070	—	N	287	287	—	6
Maryland	26,723	—	6	65	45	20	—
North Carolina	48,416	—	N	126	49	77	—
South Carolina	25,594	—	N	98	65	33	—
Virginia	33,316	—	N	144	36	108	4
West Virginia	5,139	—	N	21	11	10	—
**E.S. Central**	94,432	—	—	352	220	132	1
Alabama	29,464	—	N	144	40	104	N
Kentucky	17,134	—	N	72	55	17	N
Mississippi	17,464	—	N	49	49	—	N
Tennessee	30,370	—	N	87	76	11	1
**W.S. Central**	192,325	—	4	923	765	158	371
Arkansas	15,447	—	N	57	48	9	17
Louisiana	28,739	—	4	378	376	2	3
Oklahoma	18,278	—	N	76	30	46	N
Texas	129,861	—	N	412	311	101	351
**Mountain**	93,766	—	6,029	734	559	175	2
Arizona	30,564	—	5,861	42	33	9	—
Colorado	20,386	—	N	99	71	28	1
Idaho	5,428	—	N	147	111	36	N
Montana	3,818	—	3	125	117	8	—
Nevada	11,781	—	90	20	13	7	N
New Mexico	12,249	—	30	49	49	—	—
Utah	7,535	—	42	86	82	4	—
Wyoming	2,005	—	3	166	83	83	1
**Pacific**	218,891	1	3,277	674	378	296	2
Alaska	5,774	—	—	6	6	—	—
California	167,346	—	3,272	306	283	23	2
Hawaii	6,640	—	N	1	1	—	—
Oregon	14,181	—	5	277	35	242	—
Washington	24,950	1	N	84	53	31	—

**Territories**							
American Samoa	—	—	N	N	N	N	N
C.N.M.I.	—	—	—	—	—	—	—
Guam	937	—	1	—	—	—	—
Puerto Rico	5,969	—	N	—	—	—	—
U.S. Virgin Islands	775	—	—	—	—	—	—

	Dengue Virus Infection†		Ehrlichiosis/Anaplasmosis			
		
Area	Dengue Fever	Dengue Hemorrhagic Fever	*Anaplasma phagocytophilum*	*Ehrlichia chaffeensis*	*Ehrlichia ewingii*	Undetermined
**United States**	837	6	2,782	1,518	31	220
**New England**	34	—	747	73	1	7
Connecticut	18	—	125	—	—	—
Maine	1	—	94	3	1	2
Massachusetts	—	—	330	8	—	—
New Hampshire	4	—	88	7	—	2
Rhode Island	9	—	69	49	—	—
Vermont	2	—	41	6	—	3
**Mid. Atlantic**	206	2	591	166	1	37
New Jersey	—	—	80	50	—	2
New York (Upstate)	51	1	454	92	—	18
New York City	131	1	23	14	1	—
Pennsylvania	24	—	34	10	—	17
**E.N. Central**	65	—	705	89	—	93
Illinois	26	—	9	44	—	—
Indiana	6	—	—	—	—	48
Michigan	16	—	4	1	—	1
Ohio	9	—	4	10	—	2
Wisconsin	8	—	688	34	—	42
**W.N. Central**	38	3	660	454	20	59
Iowa	—	2	N	N	N	N
Kansas	8	—	7	86	3	—
Minnesota	21	1	630	7	—	42
Missouri	5	—	13	354	17	14
Nebraska	—	—	2	6	—	—
North Dakota	1	—	8	—	—	3
South Dakota	3	—	—	1	—	—
**S. Atlantic**	216	1	48	288	6	7
Delaware	2	—	—	14	1	1
District of Columbia	—	—	N	N	N	N
Florida	151	—	2	21	—	—
Georgia	9	—	—	20	—	—
Maryland	11	—	5	31	1	1
North Carolina	13	—	15	78	—	—
South Carolina	7	—	—	7	—	—
Virginia	21	1	23	113	4	3
West Virginia	2	—	3	4	—	2
**E.S. Central**	16	—	9	166	2	6
Alabama	5	—	3	11	—	1
Kentucky	—	—	—	67	—	—
Mississippi	1	—	1	3	—	1
Tennessee	10	—	5	85	2	4
**W.S. Central**	107	—	18	280	1	—
Arkansas	2	—	7	164	1	—
Louisiana	6	—	1	2	—	—
Oklahoma	4	—	10	106	—	—
Texas	95	—	—	8	—	—
**Mountain**	12	—	1	2	—	2
Arizona	1	—	—	—	—	2
Colorado	—	—	N	N	N	N
Idaho	1	—	N	N	N	N
Montana	5	—	—	1	—	—
Nevada	4	—	1	—	—	—
New Mexico	—	—	N	N	N	N
Utah	—	—	—	1	—	—
Wyoming	1	—	—	—	—	—
**Pacific**	143	—	3	—	—	9
Alaska	1	—	N	N	N	N
California	119	—	—	—	—	9
Hawaii	10	—	N	N	N	N
Oregon	—	—	1	—	—	—
Washington	13	—	2	—	—	—

**Territories**						
American Samoa	—	—	N	N	N	N
C.N.M.I.	—	—	—	—	—	—
Guam	—	—	N	N	N	N
Puerto Rico	9,557	153	N	N	N	N
U.S. Virgin Islands	169	5	—	—	—	—

Area	Giardiasis	Gonorrhea†	*Haemophilus influenzae*, invasive disease				Hansen’s disease (leprosy)

			All ages, serotypes	Age <5 years			

				Serotype b	Nonserotype b	Unknown serotype	
**United States**	15,106	333,004	3,792	31	222	185	81
**New England**	1,443	6,883	362	2	7	1	1
Connecticut	230	2,860	55	—	1	—	—
Maine	218	245	25	2	1	—	N
Massachusetts	664	3,106	232	—	2	—	1
New Hampshire	117	121	25	—	1	1	—
Rhode Island	41	454	13	—	—	—	—
Vermont	173	97	12	—	2	—	N
**Mid. Atlantic**	2,865	40,807	609	8	22	27	13
New Jersey	336	7,014	114	—	—	12	2
New York (Upstate)	1,010	6,460	183	2	13	1	N
New York City	764	13,459	105	—	—	12	10
Pennsylvania	755	13,874	207	6	9	2	1
**E.N. Central**	1,953	55,395	640	3	51	13	1
Illinois	311	16,464	162	1	6	1	—
Indiana	203	7,144	141	1	12	1	1
Michigan	547	10,569	102	—	9	4	—
Ohio	507	16,619	149	1	24	2	—
Wisconsin	385	4,599	86	—	—	5	—
**W.N. Central**	1,561	17,713	271	2	5	30	3
Iowa	273	1,472	1	—	—	1	1
Kansas	102	2,161	40	—	5	—	—
Minnesota	618	3,873	90	—	—	12	1
Missouri	244	7,546	95	—	—	9	—
Nebraska	169	1,385	29		—	8	1
North Dakota	44	492	13	2	—	—	N
South Dakota	111	784	3	—	—	—	—
**S. Atlantic**	2,543	73,802	945	—	25	60	19
Delaware	20	1,390	9	—	—	1	—
District of Columbia	79	2,478	12	—	—	1	—
Florida	1,114	20,818	273	—	—	22	10
Georgia	641	14,252	162	—	5	14	6
Maryland	228	5,989	102	—	8	—	2
North Carolina	N	13,666	144	—	—	19	1
South Carolina	133	7,194	106	—	8	3	—
Virginia	278	6,952	98	—	3	—	—
West Virginia	50	1,063	39	—	1	—	N
**E.S. Central**	174	25,164	259	2	15	9	5
Alabama	174	8,377	74	1	7	2	1
Kentucky	N	4,315	47	1	—	1	1
Mississippi	N	5,096	29	—	2	5	3
Tennessee	N	7,376	109	—	6	1	—
**W.S. Central**	374	51,814	195	1	16	5	16
Arkansas	119	4,007	25	—	3	—	—
Louisiana	255	8,669	52	—	—	5	—
Oklahoma	N	5,303	113	—	13	—	N
Texas	N	33,835	5	1	N	N	16
**Mountain**	1,148	15,316	328	8	55	5	3
Arizona	115	6,412	112	3	22	2	—
Colorado	355	2,820	81	—	9	—	3
Idaho	137	211	19	1	3	3	—
Montana	91	224	6	—	4	—	—
Nevada	91	2,714	14	—	—	—	—
New Mexico	99	1,918	48	1	7	—	—
Utah	228	951	42	3	10	—	—
Wyoming	32	66	6	—	—	—	—
**Pacific**	3,045	46,110	183	5	26	35	20
Alaska	82	1,128	21	2	7	—	1
California	1,991	38,166	39	—	—	31	5
Hawaii	60	718	28	—	—	4	14
Oregon	364	1,729	84	1	10	—	N
Washington	548	4,369	11	2	9	—	N

**Territories**							
American Samoa	—	—	—	—	—	—	—
C.N.M.I.	—	—	—	—	—	—	—
Guam	2	92	—	—	—	—	17
Puerto Rico	48	356	1	—	—	1	1
U.S. Virgin Islands	—	58	N	N	N	N	—

Area	Hantavirus pulmonary syndrome	Hemolytic uremic syndrome, post-diarrheal	Hepatitis, viral, acute			Hepatitis B perinatal infection

			A	B	C	
**United States**	21	329	1,781	3,050	2,138	48
**New England**	—	18	92	94	185	—
Connecticut	N	5	19	8	—	—
Maine	—	2	10	11	8	—
Massachusetts	—	5	43	71	174	—
New Hampshire	—	4	9	2	N	—
Rhode Island	—	—	4	U	U	—
Vermont	—	2	7	2	3	—
**Mid. Atlantic**	—	20	288	225	318	7
New Jersey	—	4	68	65	106	1
New York (Upstate)	—	11	75	49	115	—
New York City	—	4	92	68	16	—
Pennsylvania	—	1	53	43	81	6
**E.N. Central**	—	39	290	482	442	8
Illinois	—	5	79	94	37	—
Indiana	—	9	32	101	175	2
Michigan	—	7	83	53	74	1
Ohio	—	9	59	225	116	5
Wisconsin	—	9	37	9	40	—
**W.N. Central**	—	45	94	116	77	2
Iowa	—	6	17	11	—	—
Kansas	—	4	11	11	17	1
Minnesota	—	17	32	19	47	1
Missouri	—	13	8	61	6	—
Nebraska	—	3	13	9	2	—
North Dakota	—	2	9	—	4	—
South Dakota	—	—	4	5	1	—
**S. Atlantic**	—	40	284	884	413	5
Delaware	—	—	4	14	U	—
District of Columbia	—	—	—	—	—	—
Florida	—	14	115	323	134	2
Georgia	—	11	36	104	48	—
Maryland	—	2	29	43	53	—
North Carolina	—	7	46	75	79	1
South Carolina	—	—	14	58	—	—
Virginia	—	6	36	72	41	2
West Virginia	—	—	4	195	58	—
**E.S. Central**	—	21	59	621	354	1
Alabama	N	2	10	90	30	—
Kentucky	—	N	24	214	226	—
Mississippi	N	—	5	55	U	N
Tennessee	—	19	20	262	98	1
**W.S. Central**	4	31	146	314	117	2
Arkansas	—	3	9	50	30	—
Louisiana	1	—	14	82	19	—
Oklahoma	2	8	14	40	40	—
Texas	1	20	109	142	28	2
**Mountain**	13	33	182	106	83	2
Arizona	5	9	66	28	U	1
Colorado	2	12	51	24	21	1
Idaho	1	4	8	13	14	—
Montana	2	—	6	4	16	—
Nevada	—	1	19	29	9	—
New Mexico	3	3	20	3	12	—
Utah	—	3	12	5	11	—
Wyoming	—	1	—	—	—	—
**Pacific**	4	82	346	208	149	21
Alaska	N	N	1	1	—	—
California	3	57	255	138	72	19
Hawaii	—	4	16	4	—	—
Oregon	1	21	29	32	14	—
Washington	—	—	45	33	63	2

**Territories**						
American Samoa	N	N	—	—	—	—
C.N.M.I.	—	—	—	—	—	—
Guam	N	—	31	75	71	—
Puerto Rico	—	N	10	36	N	—
U.S. Virgin Islands	—	N	—	—	—	—

Area	Influenza-associated pediatric mortality†	Invasive Pneumococcal disease§		Legionellosis	Listeriosis	Lyme disease			Malaria
	
		All Ages	Age <5 years			Total	Confirmed	Probable	
**United States**	160	17,193	1,171	4,954	735	36,307	27,203	9,104	1,594
**New England**	8	1,220	47	363	61	12,892	9,496	3,396	130
Connecticut	—	315	16	63	22	2,925	2,111	814	20
Maine	—	121	6	23	4	1,373	1,127	246	10
Massachusetts	5	550	20	189	25	5,290	3,816	1,474	71
New Hampshire	3	101	5	27	5	1,687	1,324	363	10
Rhode Island	—	76	—	46	4	724	444	280	14
Vermont	—	57	—	15	1	893	674	219	5
**Mid. Atlantic**	19	2,293	128	1,431	179	14,139	11,278	2,861	418
New Jersey	5	598	39	241	29	3,766	2,785	981	93
New York (Upstate)	9	1,022	51	456	63	3,872	3,018	854	58
New York City	4	673	38	300	32	743	494	249	196
Pennsylvania	1	N	N	434	55	5,758	4,981	777	71
**E.N. Central**	24	3,054	187	1,311	102	2,580	2,073	507	149
Illinois	4	N	41	299	38	337	337	—	64
Indiana	4	726	36	91	11	110	101	9	20
Michigan	5	743	43	272	14	168	114	54	21
Ohio	8	1,161	43	491	24	93	74	19	33
Wisconsin	3	424	24	158	15	1,872	1,447	425	11
**W.N. Central**	12	1,043	99	183	21	2,667	1,625	1,042	109
Iowa	1	N	N	11	2	247	153	94	12
Kansas	3	149	N	17	3	34	18	16	8
Minnesota	4	536	43	49	12	2,340	1,431	909	67
Missouri	—	N	32	77	2	3	1	2	6
Nebraska	1	156	14	19	2	10	7	3	6
North Dakota	—	103	10	3	—	29	12	17	3
South Dakota	3	99	N	7	—	4	3	1	7
**S. Atlantic**	25	3,601	288	770	145	3,559	2,442	1,117	403
Delaware	1	29	1	16	1	509	400	109	9
District of Columbia	1	85	2	14	5	35	33	2	13
Florida	8	1,089	95	250	41	138	87	51	54
Georgia	4	1,138	91	66	14	8	8	—	67
Maryland	5	492	27	162	20	1,197	801	396	147
North Carolina	—	N	N	90	23	180	39	141	27
South Carolina	4	439	19	22	11	42	33	9	9
Virginia	2	N	37	123	29	1,307	925	382	75
West Virginia	—	329	16	27	1	143	116	27	2
**E.S. Central**	6	1,459	95	193	33	89	39	50	33
Alabama	1	182	18	41	5	24	11	13	2
Kentucky	2	255	13	50	11	40	17	23	9
Mississippi	1	233	18	19	4	—	—	—	3
Tennessee	2	789	46	83	13	25	11	14	19
**W.S. Central**	27	2,325	189	240	38	85	49	36	115
Arkansas	5	252	11	24	4	—	—	—	2
Louisiana	2	358	25	29	3	—	—	—	9
Oklahoma	2	N	21	19	3	3	1	2	14
Texas	18	1,715	132	168	28	82	48	34	90
**Mountain**	21	2,012	116	190	24	109	74	35	86
Arizona	4	786	44	69	3	32	22	10	33
Colorado	5	504	26	48	11	—	—	—	32
Idaho	—	N	4	12	—	19	14	5	5
Montana	—	31	1	9	—	18	16	2	—
Nevada	3	139	5	19	4	16	11	5	8
New Mexico	4	328	12	11	3	6	—	6	1
Utah	5	202	23	22	3	15	10	5	7
Wyoming	—	22	1	—	—	3	1	2	—
**Pacific**	18	186	22	273	132	187	127	60	151
Alaska	—	105	15	1	—	14	14	—	4
California	16	N	N	203	101	112	90	22	103
Hawaii	2	81	7	7	3	N	N	N	1
Oregon	—	N	N	20	7	43	12	31	13
Washington	—	N	N	42	21	18	11	7	30

**Territories**									
American Samoa	—	N	—	N	N	N	N	N	—
C.N.M.I.	—	—	—	—	—	—	—	—	—
Guam	—	18	—	—	—	—	—	—	—
Puerto Rico	1	—	—	13	—	N	N	N	—
U.S. Virgin Islands	—	—	—	—	—	N	N	N	—

	Measles			Meningococcal disease				
		
Area	Total	Indigenous	Imported	All Serogroups	Serogroups ACWY	Serogroup B	Serogroup Other	Serogroup Unknown
**United States**	187	135	52	556	142	99	17	298
**New England**	2	—	2	25	14	8	2	1
Connecticut	—	—	—	3	1	—	1	1
Maine	—	—	—	4	2	1	1	—
Massachusetts	1	—	1	11	7	4	—	—
New Hampshire	—	—	—	2	1	1	—	—
Rhode Island	1	—	1	1	1	—	—	—
Vermont	—	—	—	4	2	2	—	—
**Mid. Atlantic**	80	70	10	82	18	14	2	48
New Jersey	15	12	3	20	—	—	—	20
New York (Upstate)	3	—	3	24	10	9	1	4
New York City	62	58	4	16	—	—	—	16
Pennsylvania	—	—	—	22	8	5	1	8
**E.N. Central**	12	3	9	52	21	24	3	4
Illinois	5	—	5	10	6	3	1	—
Indiana	2	1	1	15	6	9	—	—
Michigan	5	2	3	4	2	1	—	1
Ohio	—	—	—	10	5	3	1	1
Wisconsin	—	—	—	13	2	8	1	2
**W.N. Central**	5	—	5	38	6	6	1	25
Iowa	—	—	—	1	1	—	—	—
Kansas	—	—	—	3	2	—	—	1
Minnesota	2	—	2	12	—	—	—	12
Missouri	3	—	3	10	—	—	—	10
Nebraska	—	—	—	5	—	3	—	2
North Dakota	—	—	—	3	—	2	1	—
South Dakota	—	—	—	4	3	1	—	—
**S. Atlantic**	31	27	4	98	17	9	3	69
Delaware	—	—	—	2	—	2	—	—
District of Columbia	1	1	—	—	—	—	—	—
Florida	7	5	2	58	—	—	—	58
Georgia	—	—	—	12	4	1	1	6
Maryland	1	—	1	3	1	1	—	1
North Carolina	22	21	1	10	6	3	—	1
South Carolina	—	—	—	4	3	—	1	—
Virginia	—	—	—	7	2	2	—	3
West Virginia	—	—	—	2	1	—	1	—
**E.S. Central**	—	—	—	16	6	4	1	5
Alabama	—	—	—	5	2	2	1	—
Kentucky	—	—	—	1	1	—	—	—
Mississippi	—	—	—	4	—	—	—	4
Tennessee	—	—	—	6	3	2	—	1
**W.S. Central**	27	23	4	59	23	13	—	23
Arkansas	—	—	—	7	2	4	—	1
Louisiana	—	—	—	16	—	—	—	16
Oklahoma	—	—	—	6	4	2	—	—
Texas	27	23	4	30	17	7	—	6
**Mountain**	3	1	2	40	23	9	2	6
Arizona	1	—	1	12	9	3	—	—
Colorado	2	1	1	9	5	2	—	2
Idaho	—	—	—	4	1	1	—	2
Montana	—	—	—	1	1	—	—	—
Nevada	—	—	—	1	—	—	—	1
New Mexico	—	—	—	2	1	—	1	—
Utah	—	—	—	9	5	2	1	1
Wyoming	—	—	—	2	1	1	—	—
**Pacific**	27	11	16	146	14	12	3	117
Alaska	—	—	—	—	—	—	—	—
California	17	7	10	113	—	—	—	113
Hawaii	—	—	—	1	—	—	1	—
Oregon	6	3	3	12	7	3	1	1
Washington	4	1	3	20	7	9	1	3

**Territories**								
American Samoa	—	—	—	—	—	—	—	—
C.N.M.I.	—	—	—	—	—	—	—	—
Guam	—	—	—	1	—	—	—	1
Puerto Rico	—	—	—	1	—	1	—	—
U.S. Virgin Islands	—	—	—	—	—	—	—	—

Area	Mumps	Novel influenza A virus infections†	Pertussis	Plague	Poliomyelitis, paralytic	Psittacosis	Q fever		

							Total	Acute	Chronic
**United States**	584	21	28,639	4	1	6	170	137	33
**New England**	79	—	1,147	—	—	—	2	1	1
Connecticut	4	—	61	—	—	N	—	—	—
Maine	1	—	332	—	—	—	—	—	—
Massachusetts	69	—	349	—	—	—	—	—	—
New Hampshire	1	—	131	—	—	—	N	N	N
Rhode Island	3	—	160	—	—	—	2	1	1
Vermont	1	—	114	—	—	—	N	N	N
**Mid. Atlantic**	146	—	1,903	—	—	—	7	5	2
New Jersey	80	—	406	—	—	—	2	2	—
New York (Upstate)	10	—	722	—	—	—	1	—	1
New York City	35	—	142	—	—	—	—	—	—
Pennsylvania	21	—	633	—	—	—	4	3	1
**E.N. Central**	50	18	5,111	—	—	—	25	19	6
Illinois	26	1	785	—	—	—	6	3	3
Indiana	4	14	616	—	—	—	1	1	—
Michigan	6	2	988	—	—	—	1	1	—
Ohio	12	1	1,464	—	—	—	8	6	2
Wisconsin	2	—	1,258	—	—	—	9	8	1
**W.N. Central**	15	1	2,523	—	—	1	42	35	7
Iowa	3	1	308	—	—	—	N	N	N
Kansas	—	—	405	—	—	—	3	3	—
Minnesota	3	—	865	—	—	—	2	2	—
Missouri	8	—	559	—	—	—	24	22	2
Nebraska	—	—	232	—	—	1	8	3	5
North Dakota	1	—	87	—	—	—	1	1	—
South Dakota	—	—	67	—	—	—	4	4	—
**S. Atlantic**	214	—	2,599	—	—	1	15	13	2
Delaware	—	—	57	—	—	—	—	—	—
District of Columbia	1	—	42	—	—	—	N	N	N
Florida	1	—	732	—	—	—	2	2	—
Georgia	10	—	317	—	—	—	2	2	—
Maryland	87	—	213	—	—	—	2	1	1
North Carolina	4	—	583	—	—	—	5	5	—
South Carolina	2	—	218	—	—	1	1	1	—
Virginia	109	—	418	—	—	—	3	2	1
West Virginia	—	—	19	—	—	—	—	—	—
**E.S. Central**	13	—	889	—	—	—	7	6	1
Alabama	4	—	200	—	—	—	2	2	—
Kentucky	2	—	383	—	—	—	1	—	1
Mississippi	—	—	59	—	—	—	—	—	—
Tennessee	7	—	247	—	—	—	4	4	—
**W.S. Central**	19	2	4,920	—	1	—	26	25	1
Arkansas	3	2	466	—	—	—	3	3	—
Louisiana	2	—	214	—	—	—	—	—	—
Oklahoma	1	—	255	—	—	—	3	3	—
Texas	13	—	3,985	—	1	N	20	19	1
**Mountain**	13	—	5,935	4	—	1	25	13	12
Arizona	1	—	1,440	—	—	1	8	4	4
Colorado	4	—	1,418	—	—	—	8	4	4
Idaho	—	—	237	—	—	—	2	1	1
Montana	—	—	663	—	—	—	2	1	1
Nevada	5	—	181	—	—	—	—	—	—
New Mexico	1	—	613	4	—	—	2	2	—
Utah	2	—	1,308	—	—	—	3	1	2
Wyoming	—	—	75	—	—	—	—	—	—
**Pacific**	35	—	3,612	—	—	3	21	20	1
Alaska	—	—	317	—	—	—	—	—	—
California	30	—	2,011	—	—	1	16	16	—
Hawaii	—	—	50	—	—	—	—	—	—
Oregon	3	—	486	—	—	2	3	3	—
Washington	2	—	748	—	—	—	2	1	1

**Territories**									
American Samoa	—	—	—	—	—	N	N	N	N
C.N.M.I.	—	—	—	—	—	—	—	—	—
Guam	8	—	—	—	—	—	N	N	N
Puerto Rico	3	—	34	—	—	N	—	—	—
U.S. Virgin Islands	—	—	—	—	—	—	—	—	—

Area	Rabies		Rubella	Rubella, Congenital syndrome	Salmonellosis	Shiga toxin-producing *Escherichia Coli* (STEC)†

	Animal	Human				
**United States**	4,248	2	9	1	50,634	6,663
**New England**	307	—	—	—	2,116	256
Connecticut	148	—	—	—	426	71
Maine	50	—	—	—	131	27
Massachusetts	—	—	—	—	1,143	108
New Hampshire	30	—	—	—	213	27
Rhode Island	28	—	—	—	128	3
Vermont	51	—	—	—	75	20
**Mid. Atlantic**	742	—	1	1	5,112	725
New Jersey	—	—	—	—	1,062	137
New York (Upstate)	335	—	—	1	1,298	221
New York City	56	—	1	—	1,131	88
Pennsylvania	351	—	—	—	1,621	279
**E.N. Central**	166	—	1	—	5,561	1,031
Illinois	54	—	—	—	1,783	279
Indiana	10	—	—	—	705	121
Michigan	41	—	—	—	997	184
Ohio	61	—	1	—	1,181	222
Wisconsin	N	—	—	—	895	225
**W.N. Central**	223	—	—	—	3,235	1,009
Iowa	—	—	—	—	575	171
Kansas	60	—	—	—	423	89
Minnesota	62	—	—	—	799	305
Missouri	39	—	—	—	847	276
Nebraska	34	—	—	—	307	82
North Dakota	—	—	—	—	102	43
South Dakota	28	—	—	—	182	43
**S. Atlantic**	1,238	2	—	—	13,710	549
Delaware	—	—	—	—	121	15
District of Columbia	U	—	—	—	52	5
Florida	103	—	—	—	6,133	121
Georgia	302	—	—	—	2,281	121
Maryland	376	1	—	—	862	64
North Carolina	379	1	—	—	1,877	71
South Carolina	—	—	—	—	1,139	8
Virginia	—	—	—	—	1,051	109
West Virginia	78	—	—	—	194	35
**E.S. Central**	59	—	—	—	3,397	327
Alabama	39	—	—	—	1,086	53
Kentucky	15	—	—	—	526	110
Mississippi	5	—	—	—	917	30
Tennessee	—	—	—	—	868	134
**W.S. Central**	1,180	—	1	—	7,845	813
Arkansas	152	—	—	—	706	76
Louisiana	7	—	—	—	1,282	24
Oklahoma	84	—	1	—	911	107
Texas	937	—	—	—	4,946	606
**Mountain**	108	—	3	—	3,133	791
Arizona	N	—	—	—	1,010	246
Colorado	—	—	—	—	631	186
Idaho	27	—	2	—	134	106
Montana	36	—	—	—	93	49
Nevada	13	—	—	—	522	59
New Mexico	11	—	1	—	350	30
Utah	12	—	—	—	322	84
Wyoming	9	—	—	—	71	31
**Pacific**	225	—	3	—	6,525	1,162
Alaska	7	—	—	—	87	N
California	196	—	—	—	5,043	623
Hawaii	—	—	1	—	349	28
Oregon	10	—	1	—	375	189
Washington	12	—	1	—	671	322

**Territories**						
American Samoa	U	U	—	—	—	—
C.N.M.I.	—	—	—	—	—	—
Guam	—	—	—	—	18	82
Puerto Rico	54	—	—	N	586	7
U.S. Virgin Islands	—	—	—	—	—	—

Area	Shigellosis	Spotted Fever Rickettsiosis†			Streptococcal toxic-shock syndrome	Syphilis§		
	
		Total	Confirmed	Probable		All Stages¶	Primary & Secondary	Congenital
**United States**	12,729	3,359	174	3,181	224	56,471	17,375	348
**New England**	515	9	1	8	39	1,327	502	4
Connecticut	57	—	—	—	18	133	56	—
Maine	5	2	—	2	16	21	10	—
Massachusetts	174	1	—	1	1	990	360	4
New Hampshire	8	4	1	3	—	79	28	—
Rhode Island	268	2	—	2	2	94	45	—
Vermont	3	—	—	—	2	10	3	—
**Mid. Atlantic**	871	86	4	82	37	8,626	2,163	13
New Jersey	137	42	2	40	27	968	233	—
New York (Upstate)	273	25	1	24	9	1,052	298	5
New York City	315	3	1	2	—	5,121	1,161	6
Pennsylvania	146	16	—	16	1	1,485	471	2
**E.N. Central**	1,348	171	7	164	90	5,624	2,031	49
Illinois	312	102	3	99	59	2,661	798	23
Indiana	117	32	2	30	13	543	215	—
Michigan	169	3	—	3	9	1,068	487	9
Ohio	714	23	1	22	9	1,095	436	17
Wisconsin	36	11	1	10	—	257	95	—
**W.N. Central**	870	292	11	281	1	1,753	698	3
Iowa	342	8	—	8	—	226	106	—
Kansas	40	—	—	—	—	196	51	—
Minnesota	132	15	1	14	—	541	193	—
Missouri	89	245	4	241	1	609	251	3
Nebraska	63	15	5	10	—	95	41	—
North Dakota	18	2	—	2	—	25	12	—
South Dakota	186	7	1	6	—	61	44	—
**S. Atlantic**	2,483	972	107	865	23	13,072	4,211	78
Delaware	14	11	—	11	—	146	52	1
District of Columbia	15	6	5	1	—	609	168	2
Florida	1,018	24	4	20	N	5,024	1,513	37
Georgia	886	81	81	—	—	2,990	1,017	20
Maryland	107	8	—	8	1	1,361	456	14
North Carolina	201	426	11	415	9	1,150	404	1
South Carolina	120	60	1	59	4	753	271	1
Virginia	115	350	4	346	8	1,000	315	2
West Virginia	7	6	1	5	1	39	15	—
**E.S. Central**	1,264	915	16	897	10	2,347	597	8
Alabama	313	255	1	254	—	679	183	2
Kentucky	63	72	2	68	10	395	122	4
Mississippi	222	39	1	38	N	293	78	—
Tennessee	666	549	12	537	—	980	214	2
**W.S. Central**	3,320	809	15	794	4	9,953	2,193	119
Arkansas	273	480	4	476	1	527	177	12
Louisiana	451	5	—	5	3	1,998	423	32
Oklahoma	210	241	10	231	N	383	118	—
Texas	2,386	83	1	82	N	7,045	1,475	75
**Mountain**	770	86	11	73	20	2,438	828	17
Arizona	428	63	9	54	—	962	287	13
Colorado	113	6	—	6	—	475	163	—
Idaho	11	1	1	—	—	42	15	—
Montana	69	2	—	2	—	8	5	—
Nevada	54	1	—	1	9	523	205	2
New Mexico	60	4	1	3	—	247	78	2
Utah	25	7	—	5	11	172	74	—
Wyoming	10	2	—	2	—	9	1	—
**Pacific**	1,288	19	2	17	—	11,331	4,152	57
Alaska	1	N	—	—	—	35	23	1
California	1,068	15	1	14	N	9,971	3,532	56
Hawaii	42	N	N	N	—	87	46	—
Oregon	55	2	1	1	N	527	267	—
Washington	122	2	—	2	N	711	284	—

**Territories**								
American Samoa	—	N	N	N	N	—	—	—
C.N.M.I.	—	—	—	—	—	—	—	—
Guam	7	N	N	N	—	24	6	1
Puerto Rico	4	N	N	N	N	810	385	1
U.S. Virgin Islands	—	N	N	N	—	9	2	—

Area	Tetanus	Toxic-shock syndrome	Trichinellosis	Tuberculosis†	Tularemia
**United States**	26	71	22	9,582	203
**New England**	1	—	—	325	8
Connecticut	—	N	—	62	—
Maine	1	—	—	15	—
Massachusetts	—	—	—	201	8
New Hampshire	—	—	—	15	—
Rhode Island	—	—	—	27	—
Vermont	—	—	—	5	—
**Mid. Atlantic**	1	14	2	1,405	2
New Jersey	—	3	—	319	2
New York (Upstate)	1	6	2	216	—
New York City	—	3	—	656	—
Pennsylvania	—	2	—	214	—
**E.N. Central**	2	21	9	760	12
Illinois	—	5	9	327	4
Indiana	1	—	—	94	5
Michigan	1	10	—	141	—
Ohio	—	3	—	148	2
Wisconsin	—	3	—	50	1
**W.N. Central**	2	8	1	380	92
Iowa	1	1	—	47	4
Kansas	—	—	—	36	28
Minnesota	1	6	—	151	—
Missouri	—	1	—	104	36
Nebraska	—	—	1	21	17
North Dakota	—	—	—	12	—
South Dakota	—	—	—	9	7
**S. Atlantic**	7	12	6	1,746	7
Delaware	—	—	—	19	—
District of Columbia	—	—	—	38	—
Florida	5	N	—	652	1
Georgia	—	10	N	340	—
Maryland	—	N	3	176	2
North Carolina	—	1	1	216	2
South Carolina	—	1	—	112	—
Virginia	2	N	2	180	2
West Virginia	—	—	—	13	—
**E.S. Central**	2	5	—	374	7
Alabama	—	1	—	108	—
Kentucky	1	1	N	59	3
Mississippi	—	N	—	65	—
Tennessee	1	3	—	142	4
**W.S. Central**	3	2	—	1,502	49
Arkansas	1	2	N	72	38
Louisiana	—	—	—	139	—
Oklahoma	—	N	—	69	10
Texas	2	N	—	1,222	1
**Mountain**	3	3	—	450	15
Arizona	—	1	—	184	—
Colorado	—	1	—	74	1
Idaho	1	—	—	11	3
Montana	—	—	—	6	5
Nevada	—	—	—	92	—
New Mexico	1	—	—	50	4
Utah	1	1	—	33	2
Wyoming	—	—	—	—	—
**Pacific**	5	6	4	2,640	11
Alaska	—	N	2	71	1
California	4	6	2	2,171	2
Hawaii	—	N	—	115	—
Oregon	1	N	—	73	3
Washington	—	N	—	210	5

**Territories**					
American Samoa	—	N	N	2	—
C.N.M.I.	—	—	—	16	—
Guam	—	—	—	48	—
Puerto Rico	1	N	N	50	—
U.S. Virgin Islands	—	—	—	2	—

Area	Typhoid fever	Vancomycin-intermediate *Staphylococcus aureus* (*VISA*)	Varicella		Vibriosis

			Morbidity	Mortality†	
**United States**	338	249	11,359	3	1,299
**New England**	27	—	1,116	—	179
Connecticut	5	—	223	—	41
Maine	—	—	140	—	9
Massachusetts	18	—	510	N	101
New Hampshire	3	N	104	—	4
Rhode Island	1	—	39	—	19
Vermont	—	—	100	—	5
**Mid. Atlantic**	79	54	1,204	1	162
New Jersey	30	8	419	—	56
New York (Upstate)	16	35	N	1	73
New York City	23	6	N	—	27
Pennsylvania	10	5	785	—	6
**E.N. Central**	29	37	2,834	—	50
Illinois	12	7	731	—	15
Indiana	4	—	321	—	8
Michigan	4	15	722	—	10
Ohio	5	12	661	N	11
Wisconsin	4	3	399	—	6
**W.N. Central**	14	133	1,237	1	31
Iowa	1	N	N	N	N
Kansas	2	1	434	—	1
Minnesota	7	—	478	—	19
Missouri	1	131	230	1	5
Nebraska	—	—	16	—	3
North Dakota	—	—	36	—	3
South Dakota	3	1	43	N	N
**S. Atlantic**	51	15	1,404	1	365
Delaware	1	—	23	—	7
District of Columbia	—	1	10	—	1
Florida	11	5	659	1	191
Georgia	11	—	54	—	25
Maryland	13	4	N	—	57
North Carolina	5	2	N	N	26
South Carolina	—	—	168	—	14
Virginia	10	3	374	N	42
West Virginia	—	—	116	—	2
**E.S. Central**	10	—	165	—	41
Alabama	4	—	160	—	18
Kentucky	3	N	N	N	—
Mississippi	—	—	5	N	11
Tennessee	3	—	N	—	12
**W.S. Central**	14	10	2,185	—	130
Arkansas	—	—	249	—	N
Louisiana	—	1	62	—	39
Oklahoma	1	1	N	N	7
Texas	13	8	1,874	N	84
**Mountain**	22	—	1,093	—	42
Arizona	12	—	354	—	19
Colorado	2	N	353	N	10
Idaho	2	N	N	N	N
Montana	1	—	84	—	3
Nevada	1	—	N	N	5
New Mexico	1	N	66	—	3
Utah	3	—	227	—	2
Wyoming	—	—	9	N	—
**Pacific**	92	—	121	—	299
Alaska	5	N	61	—	2
California	69	N	30	—	150
Hawaii	4	—	30	—	30
Oregon	3	N	N	N	27
Washington	11	N	N	—	90

**Territories**					
American Samoa	—	N	N	N	N
C.N.M.I.	—	—	—	—	—
Guam	—	—	57	N	1
Puerto Rico	—	2	305	—	—
U.S. Virgin Islands	—	—	—	—	—

N: Not Reportable U: Unavailable —: No reported cases C.N.M.I.: Commonwealth of the Northern Mariana Islands.

* No cases of anthrax; diphtheria; eastern equine encephalitis, nonneuroinvasive disease; poliovirus infection, nonparalytic; severe acute respiratory syndrome-associated Coronavirus disease (SARS-CoV); smallpox; St. Louis encephalitis, nonneuroinvasive disease; western equine encephalitis, neuroinvasive and nonneuroinvasive disease; yellow fever; and viral hemorrhagic fevers were reported in the United States during 2013. Data on chronic hepatitis B and hepatitis C virus infection (past or present) are not included because they are undergoing data quality review. Data for HIV diagnoses does not appear because CDC is transitioning to a new system for processing national HIV surveillance data, and will be printed in the final publication.

† Totals reported to the Division of Vector-Borne Diseases (DVBD), National Center for Emerging and Zoonotic Infectious Diseases (NCEZID) (ArboNET Surveillance), as of June 1, 2014.

§ California serogroup viral diseases for 2013 include LaCrosse encephalitis, Jamestown Canyon and California serogroup not specified.

N: Not Reportable U: Unavailable —: No reported cases C.N.M.I.: Commonwealth of the Northern Mariana Islands.

* No cases of anthrax; diphtheria; eastern equine encephalitis, nonneuroinvasive disease; poliovirus infection, nonparalytic; severe acute respiratory syndrome-associated Coronavirus disease (SARS-CoV); smallpox; St. Louis encephalitis, nonneuroinvasive disease; western equine encephalitis, neuroinvasive and nonneuroinvasive disease; yellow fever; and viral hemorrhagic fevers were reported in the United States during 2013. Data on chronic hepatitis B and hepatitis C virus infection (past or present) are not included because they are undergoing data quality review. Data for HIV diagnoses does not appear because CDC is transitioning to a new system for processing national HIV surveillance data, and will be printed in the final publication.

† Includes cases reported as wound and unspecified botulism.

§ Totals reported to the Division of STD Prevention, NCHHSTP, as of June 4, 2014.

N: Not Reportable U: Unavailable —: No reported cases C.N.M.I.: Commonwealth of the Northern Mariana Islands.

* No cases of anthrax; diphtheria; eastern equine encephalitis, nonneuroinvasive disease; poliovirus infection, nonparalytic; severe acute respiratory syndrome-associated Coronavirus disease (SARS-CoV); smallpox; St. Louis encephalitis, nonneuroinvasive disease; western equine encephalitis, neuroinvasive and nonneuroinvasive disease; yellow fever; and viral hemorrhagic fevers were reported in the United States during 2013. Data on chronic hepatitis B and hepatitis C virus infection (past or present) are not included because they are undergoing data quality review. Data for HIV diagnoses does not appear because CDC is transitioning to a new system for processing national HIV surveillance data, and will be printed in the final publication.

† Totals reported to the Division of STD Prevention, NCHHSTP, as of June 4, 2014.

N: Not Reportable U: Unavailable —: No reported cases C.N.M.I.: Commonwealth of the Northern Mariana Islands.

* No cases of anthrax; diphtheria; eastern equine encephalitis, nonneuroinvasive disease; poliovirus infection, nonparalytic; severe acute respiratory syndrome-associated Coronavirus disease (SARS-CoV); smallpox; St. Louis encephalitis, nonneuroinvasive disease; western equine encephalitis, neuroinvasive and nonneuroinvasive disease; yellow fever; and viral hemorrhagic fevers were reported in the United States during 2013. Data on chronic hepatitis B and hepatitis C virus infection (past or present) are not included because they are undergoing data quality review. Data for HIV diagnoses does not appear because CDC is transitioning to a new system for processing national HIV surveillance data, and will be printed in the final publication.

† Total number of reported laboratory-positive dengue cases including all confirmed cases [by anti-dengue virus (DENV) molecular diagnostic methods or sero-conversion of anti-DENV IgM] and all probable cases (by a single, positive anti-DENV IgM). Totals reported to the Division of Vector-Borne Diseases (DVBD), National Center for Emerging and Zoonotic Infectious Diseases (NCEZID) (ArboNET Surveillance), as of July 1, 2014.

N: Not Reportable U: Unavailable —: No reported cases C.N.M.I.: Commonwealth of the Northern Mariana Islands.

* No cases of anthrax; diphtheria; eastern equine encephalitis, nonneuroinvasive disease; poliovirus infection, nonparalytic; severe acute respiratory syndrome-associated Coronavirus disease (SARS-CoV); smallpox; St. Louis encephalitis, nonneuroinvasive disease; western equine encephalitis, neuroinvasive and nonneuroinvasive disease; yellow fever; and viral hemorrhagic fevers were reported in the United States during 2013. Data on chronic hepatitis B and hepatitis C virus infection (past or present) are not included because they are undergoing data quality review. Data for HIV diagnoses does not appear because CDC is transitioning to a new system for processing national HIV surveillance data, and will be printed in the final publication.

† Totals reported to the Division of STD Prevention, NCHHSTP, as of June 4, 2014.

N: Not Reportable U: Unavailable —: No reported cases C.N.M.I.: Commonwealth of the Northern Mariana Islands.

* No cases of anthrax; diphtheria; eastern equine encephalitis, nonneuroinvasive disease; poliovirus infection, nonparalytic; severe acute respiratory syndrome-associated Coronavirus disease (SARS-CoV); smallpox; St. Louis encephalitis, nonneuroinvasive disease; western equine encephalitis, neuroinvasive and nonneuroinvasive disease; yellow fever; and viral hemorrhagic fevers were reported in the United States during 2013. Data on chronic hepatitis B and hepatitis C virus infection (past or present) are not included because they are undergoing data quality review. Data for HIV diagnoses does not appear because CDC is transitioning to a new system for processing national HIV surveillance data, and will be printed in the final publication.

N: Not Reportable U: Unavailable —: No reported cases C.N.M.I.: Commonwealth of the Northern Mariana Islands.

* No cases of anthrax; diphtheria; eastern equine encephalitis, nonneuroinvasive disease; poliovirus infection, nonparalytic; severe acute respiratory syndrome-associated Coronavirus disease (SARS-CoV); smallpox; St. Louis encephalitis, nonneuroinvasive disease; western equine encephalitis, neuroinvasive and nonneuroinvasive disease; yellow fever; and viral hemorrhagic fevers were reported in the United States during 2013. Data on chronic hepatitis B and hepatitis C virus infection (past or present) are not included because they are undergoing data quality review. Data for HIV diagnoses does not appear because CDC is transitioning to a new system for processing national HIV surveillance data, and will be printed in the final publication.

† Totals reported to the Influenza Division, National Center for Immunization and Respiratory Diseases (NCIRD), as of December 28, 2013.

§ *Streptococcus pneumoniae*, invasive disease. Since January 1, 2010, “Invasive pneumococcal disease (IPD)” has been nationally notifiable and separate notifications for “Drug resistant *S. pneumoniae*” and “IPD in children <5 years of age” have been discontinued.

N: Not Reportable U: Unavailable —: No reported cases C.N.M.I.: Commonwealth of the Northern Mariana Islands.

* No cases of anthrax; diphtheria; eastern equine encephalitis, nonneuroinvasive disease; poliovirus infection, nonparalytic; severe acute respiratory syndrome-associated Coronavirus disease (SARS-CoV); smallpox; St. Louis encephalitis, nonneuroinvasive disease; western equine encephalitis, neuroinvasive and nonneuroinvasive disease; yellow fever; and viral hemorrhagic fevers were reported in the United States during 2013. Data on chronic hepatitis B and hepatitis C virus infection (past or present) are not included because they are undergoing data quality review. Data for HIV diagnoses does not appear because CDC is transitioning to a new system for processing national HIV surveillance data, and will be printed in the final publication.

N: Not Reportable U: Unavailable —: No reported cases C.N.M.I.: Commonwealth of the Northern Mariana Islands.

* No cases of anthrax; diphtheria; eastern equine encephalitis, nonneuroinvasive disease; poliovirus infection, nonparalytic; severe acute respiratory syndrome-associated Coronavirus disease (SARS-CoV); smallpox; St. Louis encephalitis, nonneuroinvasive disease; western equine encephalitis, neuroinvasive and nonneuroinvasive disease; yellow fever; and viral hemorrhagic fevers were reported in the United States during 2013. Data on chronic hepatitis B and hepatitis C virus infection (past or present) are not included because they are undergoing data quality review. Data for HIV diagnoses does not appear because CDC is transitioning to a new system for processing national HIV surveillance data, and will be printed in the final publication.

† Totals reported to the Influenza Division, National Center for Immunization and Respiratory Diseases (NCIRD), as of December 28, 2013.

N: Not Reportable U: Unavailable —: No reported cases C.N.M.I.: Commonwealth of the Northern Mariana Islands.

* No cases of anthrax; diphtheria; eastern equine encephalitis, nonneuroinvasive disease; poliovirus infection, nonparalytic; severe acute respiratory syndrome-associated Coronavirus disease (SARS-CoV); smallpox; St. Louis encephalitis, nonneuroinvasive disease; western equine encephalitis, neuroinvasive and nonneuroinvasive disease; yellow fever; and viral hemorrhagic fevers were reported in the United States during 2013. Data on chronic hepatitis B and hepatitis C virus infection (past or present) are not included because they are undergoing data quality review. Data for HIV diagnoses does not appear because CDC is transitioning to a new system for processing national HIV surveillance data, and will be printed in the final publication.

† Includes *E. coli* O157:H7; Shiga toxin-positive, serogroup non-O157; and Shiga toxin positive, not serogrouped.

N: Not Reportable U: Unavailable —: No reported cases C.N.M.I.: Commonwealth of the Northern Mariana Islands.

* No cases of anthrax; diphtheria; eastern equine encephalitis, nonneuroinvasive disease; poliovirus infection, nonparalytic; severe acute respiratory syndrome-associated Coronavirus disease (SARS-CoV); smallpox; St. Louis encephalitis, nonneuroinvasive disease; western equine encephalitis, neuroinvasive and nonneuroinvasive disease; yellow fever; and viral hemorrhagic fevers were reported in the United States during 2013. Data on chronic hepatitis B and hepatitis C virus infection (past or present) are not included because they are undergoing data quality review. Data for HIV diagnoses does not appear because CDC is transitioning to a new system for processing national HIV surveillance data, and will be printed in the final publication.

† Total case count includes four unknown case status reports.

§ Totals reported to the Division of STD Prevention, NCHHSTP, as of June 4, 2014.

¶ Includes the following categories: primary, secondary, latent (including early latent, late latent, and latent syphilis of unknown duration), neurosyphilis, late (including late syphilis with clinical manifestations other than neurosyphilis), and congenital syphilis.

N: Not Reportable U: Unavailable —: No reported cases C.N.M.I.: Commonwealth of the Northern Mariana Islands.

* No cases of anthrax; diphtheria; eastern equine encephalitis, nonneuroinvasive disease; poliovirus infection, nonparalytic; severe acute respiratory syndrome-associated Coronavirus disease (SARS-CoV); smallpox; St. Louis encephalitis, nonneuroinvasive disease; western equine encephalitis, neuroinvasive and nonneuroinvasive disease; yellow fever; and viral hemorrhagic fevers were reported in the United States during 2013. Data on chronic hepatitis B and hepatitis C virus infection (past or present) are not included because they are undergoing data quality review. Data for HIV diagnoses does not appear because CDC is transitioning to a new system for processing national HIV surveillance data, and will be printed in the final publication.

† Totals reported to the Division of Tuberculosis Elimination, NCHHSTP, as of July 1, 2014.

N: Not Reportable U: Unavailable —: No reported cases C.N.M.I.: Commonwealth of the Northern Mariana Islands.

* No cases of anthrax; diphtheria; eastern equine encephalitis, nonneuroinvasive disease; poliovirus infection, nonparalytic; severe acute respiratory syndrome-associated Coronavirus disease (SARS-CoV); smallpox; St. Louis encephalitis, nonneuroinvasive disease; western equine encephalitis, neuroinvasive and nonneuroinvasive disease; yellow fever; and viral hemorrhagic fevers were reported in the United States during 2013. Data on chronic hepatitis B and hepatitis C virus infection (past or present) are not included because they are undergoing data quality review. Data for HIV diagnoses does not appear because CDC is transitioning to a new system for processing national HIV surveillance data, and will be printed in the final publication.

† Totals reported to the Division of Viral Diseases, National Center for Immunization and Respiratory Diseases (NCIRD), as of May 30, 2014.
